# Splenectomy as Part of Maximal-Effort Cytoreductive Surgery in Advanced Epithelial Ovarian Cancer

**DOI:** 10.3390/cancers16040790

**Published:** 2024-02-15

**Authors:** Vasilios Pergialiotis, Eleftherios Zachariou, Vasilios Lygizos, Dimitrios Efthymios Vlachos, Emmanouil Stamatakis, Kyveli Angelou, Georgios Daskalakis, Nikolaos Thomakos, Dimitrios Haidopoulos

**Affiliations:** First Department of Obstetrics and Gynecology, Division of Gynecologic Oncology, “Alexandra” General Hospital, 115 28 Athens, Greece; elefthzach@gmail.com (E.Z.); vlygizos@gmail.com (V.L.); vlachos.dg@gmail.com (D.E.V.); emmstamatakis@hotmail.com (E.S.); kiv_ang@hotmail.com (K.A.); gdaskalakis@yahoo.com (G.D.); thomakir@hotmail.com (N.T.);

**Keywords:** splenectomy, ovarian cancer, survival, debulking surgery, cytoreduction

## Abstract

**Simple Summary:**

This retrospective study was based on outcomes of 245 women that had maximal effort cytoreduction procedures for epithelial ovarian cancer (EOC). Of those 91 had splenectomy. Comparable survival rates were observed among splenectomized and non-splenectomized patients, although, both the disease free survival (*log-rank* = 0.001) as well as the overall survival of splenectomized patients (*log-rank* = 0.006) were shorter. A significant contributor of survival rates among women having splenectomy was sepsis. Splenectomized patients offered primary debulking surgery had significantly better progression free survival compared to women receiving adjuvant chemotherapy, although the overall survival remained unaffected. The actual site of splenic metastases did not influence patients’ survival rates.

**Abstract:**

Introduction: A splenectomy is frequently performed during debulking surgery for advanced ovarian cancer. Its impact on perioperative and survival outcomes remains questionable as current evidence is conflicting. In the present study, we sought to determine the factors that affect survival rates in ovarian cancer patients that undergo a splenectomy as part of maximal-effort cytoreduction. Patients and methods: A retrospective chart review was conducted that included all epithelial ovarian cancer patients that had surgical cytoreduction for advanced epithelial ovarian cancer. Differences among splenectomized and non splenectomized patients were evaluated as well as the impact of known risk factors on survival outcomes of splenectomized patients. Results: Overall, 245 patients were identified and 223 were included in the present series, of whom 91 had a splenectomy. Recurrence rates as well as death rates were comparable among splenectomized and non-splenectomized patients; however, both the disease-free survival (*log-rank* = 0.001), as well as the overall survival of splenectomized patients (*log-rank* = 0.006), was shorter. Thrombotic events as well as rates of pulmonary embolism were comparable. Sepsis was more common among splenectomized patients. The site of splenic metastases did not influence patients’ survival. Among splenectomized patients, those offered primary debulking had longer progression-free survival (*log-rank* = 0.042), although their overall survival did not differ compared to patients submitted to interval debulking. Complete debulking significantly improved the overall survival compared to optimal debulking (*log-rank* = 0.047). Splenectomized patients that developed sepsis had worse overall survival (*log-rank* = 0.005). Discussion: The findings of our study support the feasibility of splenectomy in advanced epithelial ovarian cancer; however, its impact on patients’ survival is considerable. Therefore, every effort should be made to avoid splenic injury which will result in unintended splenectomy for non-oncological reasons.

## 1. Introduction

Ovarian cancer is the third most common gynecologic malignancy encountered in a worldwide setting, with an estimated lifetime risk of 1 in 78 women and a lifetime risk of disease-specific death of 1 in 108 women [1,2]. Major risk factors that influence the risk of ovarian cancer include older age, family history, genetic mutations, nulliparity, obesity and previous radiation exposure [3]. Early detection of the disease is rare as there are no known screening strategies; hence the majority of cases are usually referred for diagnosis in an advanced stage when the tumor load leads to the development of symptoms such as abdominal pain and bloating, difficulty to consume food and breathlessness [4]. Most cases of ovarian cancer are of epithelial origin with germ cell tumors and sex-cord tumors being involved in a small minority of cases. The distribution of ovarian cancer histology varies widely across the globe [5]; however, as the disease usually presents in advanced stage, it is considered systemic in the majority of cases; hence, its main treatment relies in the use of chemotherapy, which consists of a combination of platinum (carboplatin) and taxane (paclitaxel) chemotherapy as the primary mode of treatment [6]. In specific populations with known mutations in the breast cancer (BRCA 1 and BRCA 2) genes, as well as in those with a homologous recombination deficiency, the use of PARP (poly-ADP ribose polymerase) inhibitors in combination with anti-VEGF (vascular endothelial growth factor) therapy has achieved a significant prolongation of recurrence-free and overall survival [7]. Nevertheless, surgical treatment seems to be crucial, as complete tumor debulking significantly increases the disease-free as well as the overall survival of these patients [8]. To date, it remains unknown if primary debulking surgery offers a superior survival benefit compared to interval debulking as most of the available evidence remains conflicting and is based on studies with methodological issues in terms of the population included and the adequacy of the completion of the procedure [9,10,11].Considering that optimal debulking in primary and recurrent disease is considered the cornerstone for the success of surgical treatment and is ensured only when the tumor is completely excised, or alternatively, when the surgical goal of residual disease at a level of <1 cm is feasible [8,12]. The importance of optimal cytoreduction in the primary debulking setting was underlined by Lyons et al. who observed, in 2020, that patients submitted to primary debulking surgery with no apparent residual disease have substantially better survival outcomes compared to those receiving neoadjuvant chemotherapy [13].

Considering this evidence, surgeons searched to expand the boundaries of surgical procedures which became gradually more complex as gynecologic oncologists gained more experience and became able to perform upper abdominal surgery and accomplish multiorgan excisions. Maximal-effort cytoreductive procedures provide a significant survival benefit in ovarian cancer patients, despite the actual comorbidities, and seem to be associated with acceptable morbidity that does not necessarily hinder the use of adjuvant chemotherapy [14,15]. A splenectomy is, in our era, a rather common procedure in upper abdominal surgery for ovarian cancer as hilar and surface metastases are usually encountered in patients with advanced-stage disease that undergo primary debulking surgery [16,17]. Initially it was questioned whether the removal of the organ would negatively affect the immune system and disrupt the normal response to systemic therapy; however, novel evidence suggests that it is safe as patients do not have significantly different survival rates compared to those of patients in whom the spleen is preserved [18]. Of course, it is anticipated that a splenectomy will naturally result in an increased risk of postoperative infectious morbidity and an increased possibility of hospitalization or death from sepsis. A large population-based cohort study suggested that splenectomized cancer patients have a long-lasting increased risk of systemic inflammatory response (SIRs) that persists for years, whereas the effects of treatment for infectious morbidity in trauma patients is less likely to extend beyond the first 6 months following the procedure [19]. Similar outcomes are also observed in smaller cohorts [20], indicating that the effect of chronic diseases may be additive to that of a splenectomy and further increase the risk of chronic infectious morbidity.

It should be mentioned that, to date, it remains unknown whether the actual impact of various factors that are traditionally considered as predictive of ovarian cancer survival is significant in splenectomized patients as most of the studies that have been published in the international literature focus only on the actual impact of splenectomy on ovarian cancer survival. In the present retrospective cohort, we sought to evaluate the impact of a splenectomy on the progression-free and overall survival of ovarian cancer patients treated with high–intermediate and high-complexity-score procedures. Together we analyzed the effect of established factors that affect the survival rates of ovarian cancer patients in the splenectomized series, as to date, there is lack of substantial evidence to support a potential differential effect in this specific subgroup of patients.

## 2. Materials and Methods

### 2.1. Study Design

We retrospectively searched the medical records of all patients that underwent surgical debulking for advanced ovarian cancer between January 2016 and December 2021. We included all patients that had maximal-effort cytoreductive procedures (Surgical complexity score >4) performed by 3 ESGO accredited gynecologic oncologists. The decision to proceed to primary debulking (PDS) or interval debulking surgery (IDS) was based on tumor load and patients’ performance status. The presence of extra-abdominal metastases or extensive liver parenchymal disease that rendered hepatic excision beyond the extent of sphenoid resection necessary were considered as variables that precluded per se patients from surgical operation. The evaluation for splenic and other types of metastases was performed by computed tomography (CT) scans and, in selected cases with inconclusive results, with magnetic resonance imaging (MRI) scan. During the last 2 years, positron emission tomography (PET-CT) has also been performed in selected cases as per protocol of our institution. The performance status of patients was assessed with the Eastern Cooperative Oncology Group (ECOG) status and cases with an ECOG ≤ 3 were considered eligible for debulking procedures. The study was designed in accordance with the declaration of Helsinki for medical research involving human subjects and the institutional review board of our hospital approved this study prior to its onset (IRB approval number: 781/21).

### 2.2. Definitions

The surgical complexity of procedures was evaluated with the Mayo Clinic (Aletti) score that assigns points of surgical complexity for the following procedures: hysterectomy and bilateral salpingo-oophorectomy, omentectomy, pelvic lymphadenectomy, paraortic lymphadenectomy, pelvic peritoneal stripping, abdominal peritoneal stripping and small bowel resection. Large bowel resection, diaphragmatic stripping/resection, splenectomy and liver resection are assigned 2 points of surgical complexity. Finally, rectosigmoidectomy with reanastomosis is assigned 3 points of surgical complexity. A sum of ≤3 points indicates a low complexity score, 4–7 points an intermediate complexity score and ≥8 points a high complexity score [21].

Splenic metastases were subgrouped into hilar, surface and parenchymal considering the FIGO classification that subgroups patients into stage IVb in the presence of parenchymal disease and as stage IIIc or Iva when hilar or surface metastases are present [22]. Survival outcomes were analyzed among the different groups as current evidence for metastatic splenic disease in ovarian cancer patients does not seem to provide evidence that support its significance in recurrence rates and overall survival [23,24].

Transfusion-induced immunomodulation (TRIM) has been considered as a factor that potentially correlates with perioperative infections [25,26,27]. To date the definition of TRIM remains uncertain as there a no specific signs or symptoms and the actual incidence of the disease is unknown. To evaluate the potential severity of TRIM we subgrouped patients to three groups according to the number of intraoperative and postoperative (within 48 h) red blood cell transfusions (no transfusion, 1–2 units, >3 units).

Perioperative complications were graded using the Clavien–Dindo score and subclassified to major (Grade ≥ IIIa) and minor (Grade I–II). The timeframe of postoperative follow-up was predefined at 30 days from the operation. Major peri-operative complications were sub-categorized to direct complications that were related to the performance of splenectomy (including bleeding, hematoma formation, abscess formation and anastomotic leakage) and those usually observed in major operations (including bowel obstruction, pulmonary complications, heart failure and myocardial infarct, sepsis and deep vein thrombosis as well as pulmonary embolism). The definition of sepsis was based on the presence of confirmed infection along with at least 2 criteria of systemic inflammatory response syndrome (SIRS) which include: (i) temperature >38.3 °C or <36 °C, (ii) heart rate > 90 bpm, (iii) white blood cell count <4000 or >12,000, (iv) blood glucose > 7.7 mmol/L in non-diabetic patients or a newly diagnosed altered mental state. Minor peri-operative symptoms included lower urinary tract infections, obstructive uropathy, bladder sensory loss, lymphocyst formation and fever exceeding 38 °C.

We used the “Intraoperative Mapping of Ovarian Cancer” tool to evaluate tumor load. The instrument uses a compartmentalization of the abdomen in 9 sections to identify the extent of the presence of metastatic disease [28]. The adequacy of surgical tumor debulking was assessed using a 3-tiered system that subcategorizes patients to those that have no apparent macroscopic disease at the end of the procedure (R0 excision), those with lesion size that measures less than 1 cm (R1 excision) and those with lesion diameter that exceeds 1 cm (R2 excision).

Recurrence-free and overall survival rates were recorded from the onset of diagnosis until clinical or radiology findings of disease relapse or until patient death, respectively. For cases that had been clinically evaluated during the last 30 days, patient records were used to document disease relapse and/or death, whereas for the remainder of patients, information on survival was retrieved by direct phone calls.

#### Evaluated Outcomes

Cases that had splenectomy were compared to those that did not have one in terms of baseline patient characteristics, overall and progression-free survival outcomes and perioperative morbidity.

The independent characteristics that influenced splenectomized patients’ survival were also investigated, including the site of splenic metastases, the setting of the operation (PDS vs. IDS), the complexity of the surgical procedure (intermediate vs. high) and the presence of postoperative residual disease, as well as the presence of postoperative sepsis.

### 2.3. Statistical Analysis

Statistical analysis was performed using the SPSS 20.0 program (IBM Corp. Released 2011. IBM SPSS Statistics for Windows, Version 20.0. Armonk, NY, USA: IBM Corp.). Evaluation of the normality of distributions was performed with graphical methods and the Kolmogorov–Smirnoff analysis. The differences in continuous variables were assessed using the Mann–Whitney and Kruskal–Wallis tests (due to the abnormal distribution that was observed during the evaluation of normality), whereas dichotomous variables were analyzed with the chi-square test. Fisher’s exact test was applied wherever the number of observations was lower than five in the case of dichotomous variables. The Kaplan–Meier method was carried out to perform survival analyses. The level of significance for all analyses was set to *p <* 0.05.

## 3. Results

### 3.1. Whole Cohort

Overall, 245 cases of ovarian cancer that had maximal-effort procedures were retrieved from the medical records of our institution. Of those, 22 cases were excluded due to the lack of complete data referring to the progression free and overall survival. Two hundred and twenty-three women were included in the present study with a median age of 63 (22–86) years. Overall, 91 cases had splenectomy, whereas the remainder had maximal-effort debulking surgery that did not include a splenectomy. Comparable baseline characteristics were noted among patients that had a splenectomy and those that did not, with the exception of age which was significantly lower in patients undergoing a splenectomy (Table 1).

**Figure 1:**
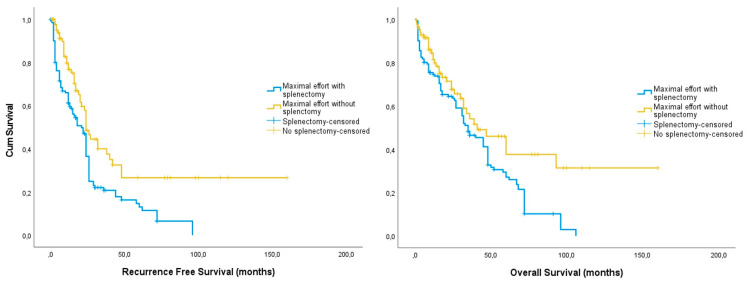
Kaplan–Meier curves of recurrence free (**left**) and overall (**right**) survival among splenectomized (blue) and non-splenectomized (yellow) epithelial ovarian cancer patients undergoing maximal-effort debulking surgery.

The absolute rates of recurrences and deaths were comparable among splenectomized patients and those who had maximal-effort surgery without a splenectomy; however, the disease-free survival was significantly shorter in splenectomized women (21 months (17.22, 24.78) vs. 24 months (20.20, 27.80), *log-rank* = 0.001) (Figure 1). Similar results were observed in the overall survival of splenectomized patients (35 months (26.17, 43.83) vs. 41 months (21.95, 60.05), *log-rank* = 0.006). After omitting cases offered secondary debulking surgery, we observed that the differences in disease-free survival remained shorter in the splenectomy group (27 months (18.22, 35.78) vs. 60 months (40.00, 80.07, *log-rank* = 0.007) and similar results were obtained for overall survival (39 months (24.32, 53.68) vs. 67.74 (47.63, 87.8).

Deep venous thrombosis and pulmonary embolisms were comparable among the two groups. Similarly, infectious diseases and surgical site infections did not differ. It should be noted, however, that cases offered a splenectomy had significantly higher rates of septic events compared to those that did not have a splenectomy (20.9% vs. 9.8%).

### 3.2. Splenectomy Cases Only

Among splenectomized patients, high-grade serous carcinoma comprised the majority of cases (seventy-nine women) whereas the remaining cases referred to other histological subtypes. Fifty-three women were diagnosed with stage IIIc disease, whereas the remaining thirty-eight cases were diagnosed with stage IV disease, primarily due to parenchymal splenic metastases (thirty-four cases). Patients’ ECOG status varied from 0 to 2 with the majority involving women that were fully active (seventy-three cases) and the remaining eighteen cases referring to either restriction in strenuous activity (twelve cases) or ECOG performance status two patients (six cases). Concerning comorbidities, thirty cases were diagnosed with hypertensive disorders, thirteen cases with cardiac disease, six cases with chronic obstructive pulmonary disease, 8 cases with autoimmune disorders and thirteen cases with diabetes mellitus.

Hilar metastases were only observed in 19 patients, surface only metastases were observed in 28 patients and parenchymal only metastases were noted in 30 patients. There were no differences in the progression free survival of patients according to the metastatic site (*log-rank* = 0.303), even after omitting cases that had a secondary debulking surgery (*log-rank* = 0.193). Similarly, the overall survival of the three groups was comparable (*log-rank* = 0.822) as well as that of patients submitted to primary debulking compared to interval debulking surgery (*log-rank* = 0.102).

Primary debulking surgery comprised the majority of cases, accounting for 59 patients, followed by those submitted to IDS (17 cases) and secondary debulking surgery (14 cases). The median duration of the performed procedure was 260 min ranging from 240 up to 320 min, with a median estimated blood loss of 600 mL (200–1500 mL). Patients submitted to a splenectomy had significantly longer procedures (290 (240–380) min) compared to patients that did not have a splenectomy (240 (240–310) min. Sixty patients (66%) had a high-complexity-score procedure, whereas the remaining patients had an intermediate-complexity procedure. Seventy-seven patients had a complete tumor debulking, whereas fourteen patients had clinically detectable disease < 1 cm.

**Figure 2:**
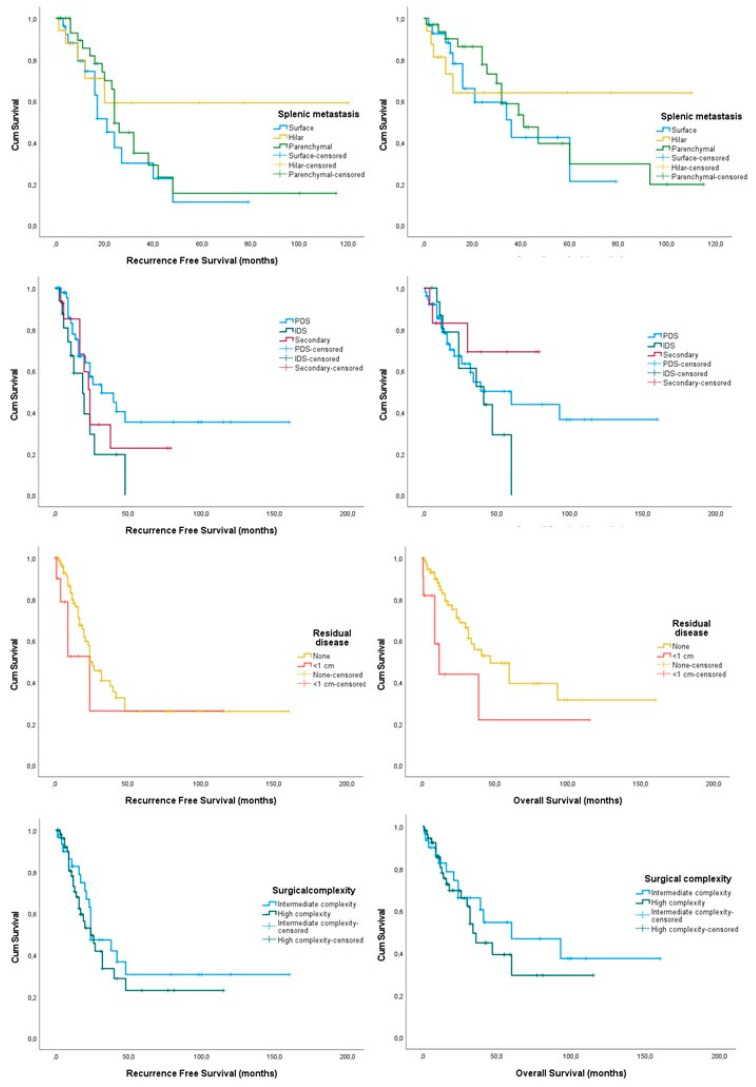
Kaplan–Meier curves of recurrence free (**left**) and overall (**right**) survival rates among selected subgroups of splenectomized epithelial ovarian cancer patients undergoing maximal-effort debulking surgery.

The median progression-free survival of splenectomized patients was 40 months ranging between 22 and 57 months, whereas the median overall survival was 73 months ranging between 53 and 92 months. In terms of survival outcomes, we observed that the site of splenic metastases did not influence the recurrence-free and overall survival of patients included (*log-rank*= 0.313 and 0.822, respectively) (Figure 2, Table 2). Similar results were obtained after comparing patients submitted to primary debulking surgery with patients that had interval debulking surgery (*log-rank* = 0.193 for progression free survival and 0.652 for overall survival). On the other hand, patients offered primary debulking surgery had a considerably better recurrence-free survival rate, although the overall survival rates did not differ significantly (*log-rank* = 0.042 and 0.519, respectively). These were not, however, considerably different compared to those of patients offered interval debulking surgery (*log-rank* = 0.102 for progression free survival and *log-rank* = 0.472 for overall survival).

Patients with optimal tumor debulking developed recurrences earlier, compared to those with complete tumor debulking, although the difference was not statistically significant (*log-rank* = 0.259); however, the extent of tumor resection was significantly associated with the patients’ overall survival (*log-rank* = 0.047). Excluding patients submitted to secondary debulking surgery, we observed that progression-free and overall survival differences were comparable, irrespective of the adequacy of the procedure (*log-rank* = 0.852 for progression free survival and 0.181 for overall survival).

In terms of surgical characteristics, we observed that patients offered high-surgical-complexity procedures had a non-statistically significant reduction in survival outcomes (*log-rank* = 0.413 for PFS and 0.446 for OS) which remained non-significant even after the omission of cases submitted to secondary debulking surgery (*log-rank* = 0.350 and 0.777, respectively). Moreover, intraoperative complications were more prevalent as the median blood loss in high-complexity procedures was considerably higher (675 mL (200–1500) vs. 500 mL (150–800) *p* = 0.004) as well as the possibility of intraoperative red blood cell (1 unit (0–3) vs. 3 units (1–7) *p* = 0.001) and plasma transfusion (1 unit (0–3) vs. 2 units (0–7), *p* = 0.007). These results remained consistently significantly different among patients submitted to PDS compared to those that had neoadjuvant chemotherapy (700 mL (200–1500) vs. 500 mL (200–1100) *p* = 0.003 for blood loss), (2 (0–4) 1 (0–1) *p* = 0.048 for intraoperative blood cells) and (2 (1–5) vs. 0 (0–2) *p* = 0.012 for plasma transfusions). Differences in blood transfusions in the postoperative group did not differ among the two groups (0 units (0–2) vs. 0 units (0–4), *p* = 0.163).

Seven surgical site infections were noted during the postoperative period, including two cases with cellulitis and five cases requiring surgical debridement due to tissue necrosis. Septic events were noted in 19 patients and deep vein thrombosis and pulmonary embolism in three cases. Septic events did not influence the progression-free survival of patients (*log-rank* = 0.200) but had a detrimental impact on the overall survival (*log-rank* = 0.005). This effect remained evident even after the omission of cases submitted to secondary debulking surgery (*log-rank* = 0.675 for PFS and *log-rank* = 0.049 for OS, respectively). Pancreatic leaks were observed in seven patients. All of them were treated conservatively with the use of CT-guided drainage. Among those, two patients required drainage for more than 2 weeks.

Concerning the impact of transfusion on survival outcomes of splenectomized patients we observed that women that did not receive transfusion had improved overall survival rates compared to those that did, although differences were not significant (*log-rank* = 0.224). After excluding cases that had secondary debulking surgery, we did not observe significant differences among PDS and IDS patients (*log-rank* = 0.651). Recurrence-free survival was comparable to that of patients that received transfusion (*log-rank* = 0.571), even after the omission of patients that had secondary debulking for disease relapse (*log-rank* = 0.544). Subgroup analysis according to the predetermined cut-off number of transfused red blood cell units (no transfusion, 1–2 units, >3 units) did not reveal any differences in recurrence-free (*log-rank* = 0.695) or overall survival (*log-rank* = 0.416).

## 4. Discussion

### 4.1. Study Findings

The findings of our study suggest that splenectomy is feasible during debulking surgery for ovarian cancer, as the rates of perioperative complications are comparable among splenectomized and non-splenectomized patients. It should be noted, however, that the rates of septic adverse events were increased, and these may influence the overall survival of splenectomized patients. The site of involvement of splenic metastases does not have a significant effect on survival outcomes of these patients; however, PDS and complete surgical excision significantly increase the possibility of prolonged survival compared to IDS and optimal (<1 cm).

### 4.2. Comparison with Existing Knowledge

Splenectomy has been considered as part of debulking surgery for the treatment of ovarian cancer for at least 3 decades and the findings of published studies seem to confirm the slightly higher postoperative infectious morbidity, including sepsis which seems to be within clinically acceptable rates, considering the benefit of complete resection among those that present with splenic metastases [17,29,30].

Its impact on patient survival seems to be questionable as several smaller studies suggest that splenectomized patients do not experience differences in disease-free survival or overall survival [17,31]. In our series, despite the comparable rates of disease recurrences and overall deaths among cases requiring splenectomy and those that did not, we observed that the interval to recurrence and death was significantly shorter. It is important to note that this effect does not seem to be significant among cases that relapse early during the course of the disease (Gehan–Breslow for early recurrences = 0.428), but is of particular importance among cases that recur at a later stage (Tarone–Ware for late recurrences < 0.001). Similar results are observed in the overall survival of these patients (Gehan–Breslow for early recurrences = 0.633 vs. Tarone–Ware for late recurrences *<* 0.001). It seems, therefore, that splenectomy mostly affects patients that are more likely to respond adequately to chemotherapy but relapse in a later stage. The actual pathophysiologic rationale behind this observation remains unclear, although the immunosuppressive effect of a splenectomy should be considered. To date, only scarce, outdated evidence exists concerning the actual effect of a splenectomy on tumor growth and anti-tumor immune system and these seem to be conflicting regarding its role in cancer progression and patient survival [32,33,34,35,36]. It should be noted, however, that population-based studies that evaluated the long-term risk of splenectomy in cancer survivors observed an increased risk of late-infection related mortality, something that could potentially explain our findings too [37,38]. It remains unknown whether infectious morbidity is the actual reason for reduced survival, or if the long-term increased likelihood of infections has a detrimental impact on cancer relapse rates, thus resulting in increased rates of reduced cancer specific survival rates. To date, the pathophysiologic pathways that connect cancer relapse to chronic inflammation have been extensively studied and the evidence indicates that a positive association seems to exist [39]. In colorectal cancer, postoperative infections seem to directly affect patients’ overall survival [40]. In gynecological cancer, evidence is still lacking, hence the assumption is based only on an extrapolation of findings in other cancer fields.

In our series, for the first time we indicated, in a large cohort of patients, that, despite the comparable rates of infectious diseases, the rates of postoperative sepsis are increased in ovarian cancer patients undergoing a splenectomy and these directly influence patients’ survival. It should be noted that this outcome does not reflect only early recurrences, but also extends to the group with an extended survival beyond 48 months as all the methods that were used to estimate differences in survival distributions revealed statistically significant differences (Gehan–Breslow for early recurrences = 0.003, log-rank for equal weight to all time points = 0.005 and Tarone–Ware for late recurrences = 0.004). It is unclear why this observation occurs; however, similar results have been reported in patients experiencing postoperative sepsis in other cancers as well [41]. One could assume that this effect might be the result of delayed access to adjuvant treatment following surgery [42] and this could explain early recurrences and deaths. However, for women that have a survival that extends beyond 3 years, it can be speculated that the effect of splenectomy might extend beyond the immediate postoperative period as chronic infections have been already described as a major cause of death among cancer patients [43].

The actual site of splenic metastases did not have an impact on survival rates of ovarian cancer patients in our cohort. This finding contrasts with those of recent studies that support that hematogenous splenic metastases (parenchymal disease) should be considered as a negative prognostic factor [44,45]. It should be noted that, despite the inability to detect considerable differences among the three groups of patients, graphic representation of the disease free survival and overall survival with the Kaplan–Meier-derived graphs (Figure 2) suggests the existence of potential differences among patients with hilar metastases compared to those with surface and parenchymal disease, an observation that indicates the need for larger sample sizes to accurately predict the importance of this variable on patients survival rates. Nevertheless, considering the potential chemotherapy resistance of parenchymal splenic disease that has been assumed by Spencer et al., and given the absence of novel evidence, it is important to stress the importance of splenectomy in this group of patients, as it is of paramount importance for the extension of the survival of patients [46].

### 4.3. Strengths and Limitations

Our study is based on one of the largest cohorts of patients which is derived from a single institution that is accredited by ESGO as a center of excellence for the management of patients with ovarian cancer. In this series, for the first time we indicate the potential variables that may affect the survival rates of ovarian cancer patients that have a splenectomy as part of their debulking surgery. The impact of infectious morbidity on survival outcomes of splenectomized patients is also denoted for the first time in the international literature, and the long-term effect of postoperative sepsis is in accordance with the findings of larger cohort studies that rely on population-based databases of cancer survivors. The rates of splenectomy seem to be quite high in the present series, accounting for 37% of included cases, whereas previous studies from large institutions indicate that the procedure is required in approximately 20% of cases with an intermediate or high complexity score [47]. Although we cannot quite explain this discrepancy, we believe that this may be attributed to the fact that our gynecologic oncology unit is the largest one in Greece, handling complex operations that are usually referred from other secondary and tertiary centers.

Despite the use of a continuous series of patients, the retrospective design of our study cannot completely preclude the possibility of bias. The lack of a substantial number of controls, which is extremely hard to obtain in single-institutional studies, rendered the use of propensity score matching impossible, which minimizes the effect of confounding factors on the outcome of interest, as the technique requires a larger cohort of unselected control cases to derive an adequately matched control group [48]. Moreover, the retrospective nature of the study precluded the investigation of the specific cause of death of included patients; therefore, the actual cancer-specific survival remains unknown, as well as the absolute rates of severe long-term infections that might directly influence the course of the disease. Lastly, the effect of BRCA and HRD status, as well as the impact of PARP inhibitors, was not evaluated as the cohort covered a wide range of years, including the pre-PARP period; hence, further research is required to evaluate if a different effect will be observed in future cohorts.

### 4.4. Implications and Conclusions

A splenectomy should be considered feasible during debulking surgery for the treatment of ovarian cancer. It directly impacts patients’ survival; however, this effect seems to be more pronounced among cases that tend to relapse later during the course of the disease. In line with current knowledge derived from patients with advanced-stage disease, survival rates of splenectomized patients are directly affected by the timing of the operation (primary debulking is superior to interval debulking surgery) and the presence of residual disease (complete resection is superior compared to optimal resection). Postoperative infectious morbidity does not increase, with the exception of sepsis which is expected to be encountered in approximately 20% of patients; however, the impact of sepsis on patients’ overall survival is significant and refers both to patients that experience early relapse and death as well as those that experience recurrence and die later in the course of the disease. To date it remains unknown if the bacterial pathogens that are the cause of postoperative infectious morbidity differ among non-splenectomized patients and those that have a splenectomy, and if antibiotic therapy needs to be more aggressive in the latter group. Considering this information, it becomes evident that every effort should be made to avoid unintended splenic injury during upper abdominal surgery as its preservation seems to be desirable in the absence of metastatic disease. In cases of minor injury, interventions to control bleeding with absorbable hemostatic agents should be carried out before a decision is made to proceed with splenectomy. Further research is needed to determine if these patients experience recurrent infections at considerably higher rates compared to patients that do not have splenectomy and if this has an impact on their survival.

## Figures and Tables

**Table 1:** Baseline characteristics among spenectomized epithelial ovarian cancer patients and non-splenectomized epithelial ovarian cancer patients. ECOG: Eastern Cooperative Oncology Group; DVT: deep vein thrombosis.

Comparison of Baseline Characteristics and Postoperative Outcome			
Baseline Characteristics			
Variable	Splenectomy	Control	*p*-Value
Age	61 (32–78)	67 (22–81)	0.041
BMI	30 (18–34)	32 (24–34)	0.238
ECOG status ECOG 0 ECOG 1 ECOG 2	7/91 12/91 72/91	4/132 17/132 111/132	0.281
Stage IIIc IV	41/74 33/74	62/104 42/104	0.575
Histology Serous Other	72/91 18/91	108/132 24/132	0.734
Operation timing PDS IDS Secondary	60/91 14/91 17/91	82/132 20/132 28/132	0.819
Residual disease None <1 cm	80/91 10/91	102/130 28/130	0.443
**Postoperative outcomes**			
DVT	3/91	5/132	0.846
Pulmonary embolism	3/91	3/132	0.642
Infectious diseases	26/91	34/132	0.641
Surgical site infection	7/91	17/132	0.219
Sepsis	19/91	13/132	0.021
Recurrences	43/91	74/132	0.196
Deaths	35/91	61/132	0.251

**Table 2:** Progression free and overall survival among splenectomized patients with preselected known factors that affect survival rates in epithelial ovarian cancer.

Factors Affecting Patient Survival				
	Progression Free Survival		Overall Survival	
Factor	Months (95% CI)	*p*-Value	Months (95% CI)	*p*-Value
Stage IIIc IV	40.00 (25.64, 54.35) 24.00 (22.89, 25.17)	0.384	60.00 (11.58, 37.30) 41.00 (26.59, 55.41)	0.820
Splenic metastases Surface Hilar Parenchymal	28.13 (17.06, 39.19) 103.33 (73.51, 133.154) 39.33 (24.05, 54.62)	0.313	40.53 (26.80, 54.29) 72.73 (45.87, 99.56) 54.94 (37.62, 72.27)	0.822
Operation setting PDS IDS	69.48 (45.25, 93.71) 23.12 (13.88, 32.35)	0.047	78.34 (53.49, 103.19) 37.67 (26.70, 48.63)	0.047
Tumor resection Complete Optimal (<1 cm)	58.94 (40.48, 77.40) 49.77 (4.00, 98.67)	0.259	73.80 (52.77, 94.84) 41.92 (5.26, 78.58)	0.047
Surgical complexity High Intermediate	42.48 (27.04, 57.92) 66.92 (39.77, 94.06)	0.413	53.41 (36.36, 70.47) 82.34 (53.68, 110.99)	0.446
Postoperative sepsis Present Absent	24.00 (16.26, 31.74) 26.00 (7.57, 44.23)	0.200	26.00 (7.74, 52.94) 60.00 (34.06, 85.94)	0.005

## Data Availability

Data available upon reasonable request.
